# Supplementary figures and images for: A spinal origin for the obligate flexor synergy in the non-human primate: Implications for control of reaching
Source: bioRxiv. 2025 Jul 30:2025.07.28.666086. Preprint. [Version 1] doi: 10.1101/2025.07.28.666086 (PMC12324299; doi:10.1101/2025.07.28.666086)

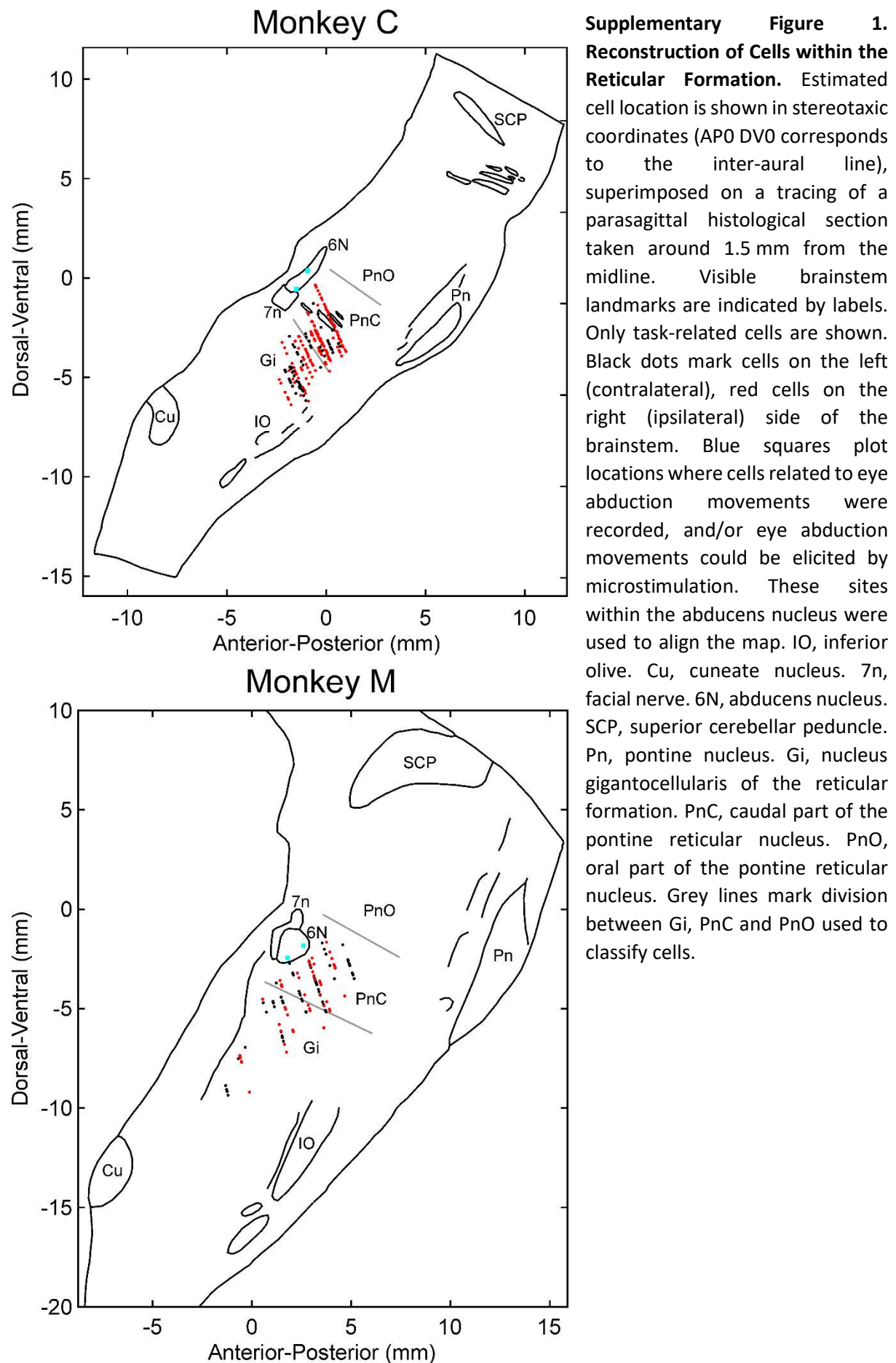

Supplement: Supplement 1 [file NIHPP2025.07.28.666086v1-supplement-1.pdf]
